# Biochemical biomarkers of knee osteoarthritis progression: Results from the FNIH biomarkers consortium progress OA study

**DOI:** 10.1016/j.ocarto.2025.100677

**Published:** 2025-09-05

**Authors:** Jamie E. Collins, Douglas Robinson, Nigel Arden, Anne Christine Bay-Jensen, Leticia A. Deveza, Morten Karsdal, Christoph Ladel, Thomas A. Perry, Christopher J. Swearingen, David J. Hunter, Virginia B. Kraus

**Affiliations:** a Brigham and Women's Hospital, Boston, MA, United States; b EMD Serono, Billerica, MA, United States; c Nuffield Department of Orthopaedics, Rheumatology and Musculoskeletal Sciences (NDORMS), University of Oxford, United States; d Nordic Bioscience A/S, Herlev, Denmark; e Sydney Musculoskeletal Health, Kolling Institute, University of Sydney, Sydney, NSW, Australia; f CHL4special Consultancy, Darmstadt, Germany; g Centre for OA Pathogenesis Versus Arthritis, Kennedy Institute of Rheumatology, Oxford University, Oxford, United Kingdom; h Biometrics, Biosplice Therapeutics, Inc., San Diego, CA, United States; i Duke Molecular Physiology Institute, Department of Medicine, Duke University School of Medicine, Durham, NC, United States

**Keywords:** Biochemical biomarkers, Prognostic modeling, Randomized controlled trials

## Abstract

**Objective:**

The Foundation for National Institutes of Health (FNIH) OA Biomarkers Consortium aims to identify and qualify biomarkers to support drug development in knee osteoarthritis (OA). The PROGRESS OA study aims to externally validate prognostic biomarkers identified in the earlier Phase I study.

**Design:**

PROGRESS OA included data from the control arms of several completed randomized controlled trials (RCTs) for knee OA. The primary outcome was medial or lateral joint space width loss (JSWL) ≥0.7 ​mm. Secondary outcomes included medial or lateral JSWL combined with symptomatic progression. Nine biochemical biomarkers identified in Phase I were included here. Logistic regression with elastic net selection examined associations between baseline biomarkers and outcomes over 12–36 months, separately for subgroups with available serum, urine, and both serum and urine biomarkers.

**Results:**

In total, the study included 827 participants across three RCTs, 159 (19 ​%) with JSWL≥0.7 ​mm. In 681 participants with both serum and urine biospecimens, the combination of serum hyaluronan (HA), urinary C2C-HUSA, body mass index (BMI), and Kellgren-Lawrence grade (KLG) yielded AUC 0.627 (95 ​% CI: 0.573, 0.681) for predicting JSWL≥0.7 ​mm, compared to AUC 0.612 (95 ​% CI: 0.557, 0.667) for BMI and KLG alone. Serum (HA) and urinary C2C-HUSA were consistently selected as predictors of disease progression in elastic net models across the subgroups.

**Conclusions:**

Biomarker associations were consistent with Phase I findings, but predictive performance remained modest. Future work in the FNIH OA Biomarkers consortium project will focus on evaluating short term changes in biomarkers to assess their potential for monitoring treatment efficacy.

## 1 Introduction

Knee osteoarthritis (OA) is a common and disabling joint disease that affects an estimated 14 million people in the U.S. and 500 million worldwide [1,2]. The disease is characterized by pain, aching, and stiffness and is the leading source of disability in people older than 55 years of age [3]. OA was ranked as the 11th leading cause of global disability among 291 conditions [4].

Drug development in OA has been challenging, and there are currently no disease-modifying OA drugs (DMOADs) approved [5]. One challenge in OA clinical trials is the relatively slow progression of the disease in unselected populations; subsequently, DMOAD clinical trials must either be very large or involve extended follow-up periods to have adequate statistical power to show a treatment effect when one truly exists [[6], [7], [8]].

Prognostic biomarkers are used “to identify the likelihood of a clinical event, disease recurrence or progression in patients who have the disease or medical condition of interest” and may be used for trial enrichment [9,10]. Prognostic enrichment involves selecting patients who are more likely to experience the clinical event of interest, such as OA progression. Prognostic biomarkers are not expected to predict treatment response, but rather to identify patients at high risk of outcomes; according to guidance from the US Food and Drug Administration, prognostic enrichment “would increase the absolute effect difference between groups but would not be expected to alter relative effect.” [11] Such biomarkers would enable the enrichment of progressors in DMOAD trials, potentially leading to more efficient trials (e.g., smaller sample sizes, higher power, lower cost). The idea underpinning prognostic enrichment is that a higher event rate in an “enriched” cohort will lead to more individuals at risk for the trial outcomes resulting in a smaller sample size needed to show a treatment effect, thus allowing for smaller and more efficient trials [[11], [12], [13]].

The Foundation for National Institutes of Health (FNIH) OA Biomarkers Consortium aims to identify and qualify biomarkers to support new drug development in OA [14]. Phase I of the project was a nested case-control study using data from the Osteoarthritis Initiative (OAI) that sought to determine the predictive ability of 18 serum and urine investigative biomarkers fulfilling one or more additional BIPEDS (burden of disease, investigative, prognostic, efficacy of intervention, diagnostic, safety) criteria for knee OA [15]. Phase I identified nine biomarkers with modest predictive ability for clinically relevant knee OA progression in the OAI cohort. The findings of Phase I informed the design of the Phase II PROGRESS OA project, reported here, in which we aimed to further qualify these biochemical biomarkers in the placebo arms of existing completed OA clinical trials [16].

## 2 Methods

### 2.1 Study design

The PROGRESS OA study includes data from the placebo arms of several completed randomized controlled trials (RCTs) that tested various therapeutic interventions for knee OA. Three trials that collected baseline serum and/or urine biospecimens were included in this analysis. Two trials assessed the effect of oral salmon calcitonin on 24-month change in joint space width (JSW) and on knee pain and function using the Western Ontario and McMaster Universities Osteoarthritis (WOMAC) questionnaire (Novartis CSMC021C2301, NCT00486434 (Nordic 2301); CSMC021C2302, NCT00704847 (Nordic 2302)) [17]. Study inclusion criteria included confirmed radiographic knee OA of Kellgren-Lawrence grade (KLG) 2 or 3 and pain in the index knee ranging between 6 and 12 on the WOMAC Pain subscale (0–20, 20 worst). The third trial, VIDEO (Vitamin D Effect on Osteoarthritis, ISRCTN 94818153), evaluated the effect of vitamin D supplementation on knee OA progression in participants with knee pain and radiological OA in the medial tibio-femoral knee compartment (modified KLG 2 or 3, JSW >1 ​mm) [18]. Participants in each study provided signed informed consent, and all studies were approved by local ethics committee or institutional review board.

We harmonized the data from these three trials. For this analysis, we included participants with baseline and follow-up JSW measurements, with baseline KLG 2 or 3, and with baseline medial and/or lateral minimum JSW >0.7 ​mm. Serum and urine biospecimens were not collected in all trials; within trials, not all participants had complete serum and urine biomarker data. Therefore, all statistical analyses were conducted in each of the three sub-cohorts: (1) participants with urine biomarkers, (2) participants with serum biomarkers, and (3) participants with both serum and urine biomarkers.

### 2.2 Outcomes

The primary outcome was joint-space width loss (JSWL) ≥0.7 ​mm from baseline to up to 36 months follow-up in either the lateral or medial compartment. Secondary outcomes included knee pain progression, defined as an increase of ≥9 points on the WOMAC pain subscale (0–100 scale, higher score representing worse pain), JSWL ≥0.5 ​mm, and the composite outcome of JSWL ≥0.7 ​mm and knee pain progression. Additionally, we examined JSWL in the medial and lateral compartments separately.

### 2.3 Biochemical biomarkers

The biomarkers chosen for this analysis all demonstrated an ability to predict clinically relevant progression, singly or in combination, as described in the Phase 1 study [15]. Details are provided in Supplementary Table S1. Urine biomarkers were analyzed both with and without normalization to urinary creatinine. Details on technical performance are provided in Supplementary Table S2.

### 2.4 Statistical analysis

Biomarkers were transformed using natural logarithms prior to analysis, then standardized to z-scores. Pearson correlation was used to assess correlations between biomarkers. Associations between biochemical biomarker concentration and progression outcomes were first assessed using univariate logistic regression. Due to high correlation between biomarkers, elastic net with 5-fold cross-validation was used to fit a penalized logistic regression model in order to determine the combination of biomarkers that best predicted progression (maximized Area Under the Curve (AUC)) [19]. We ran elastic net on 1000 bootstrap samples and retained biomarkers and covariates selected in >80 ​% of samples. We ran models that included urine markers only (with and without normalization to urinary creatinine), serum markers only, and urine and serum markers together. Elastic net models were run with and without the baseline covariates age, sex, BMI, and baseline KLG. AUCs with 5-fold cross-validation were computed for models including the selected set of covariates.

As part of the exploratory analysis, we calculated the optimal cut-point for each biomarker to maximize the AUC for predictive accuracy using Youden's J statistic [20]. Cut-points were selected using bootstrapping with 1000 replicates. We present the AUC, sensitivity, and specificity for each cut-point. Analyses were conducted using the *cutpointr* package using bootstrapping with 1000 replicates [21]. The threshold for an acceptable Youden's J statistic for a diagnostic test is generally considered to be ​≥ ​50 (e.g., a sensitivity and specificity of both ≥75 would meet this threshold) [22].

Analyses were run using R Studio (version 2024.9.1.394) using the packages caret (v 6.0.94), glmnet (v 4.1.8), and cutpointr (1.1.2) [21,23,24].

## 3 Results

A total of 598, 509, and 237 participants were randomized to the placebo arms of Nordic 2301, Nordic 2302, and VIDEO trials, respectively [6,18]; of these, 483, 312, and 49 participants had biochemical biomarker data and baseline and follow-up JSW measures, respectively. A further seventeen participants were excluded from VIDEO after KLG and baseline JSW exclusions, resulting in a total analytic sample of 827. A total of 722 (87.3 ​%) participants had serum biomarkers, 786 (95.0 ​%) had urine biomarkers, and 681 (82.4 ​%) had both; these participants comprised the three groups analyzed. Of the 827 study participants included for analysis, 65 ​% were female with a mean age of 65 years (SD 6.6) and a mean body mass index (BMI) of 29 ​kg/m^2^ (SD 4.8) (Table 1). A total of 83 ​% of knees were KLG 2 and 17 ​% were KLG 3. The primary outcome of JSWL of ≥0.70 ​mm in any joint compartment (medial or lateral) was achieved by 159 (19 ​%) participants over the course of follow-up; six (1 ​%) had both medial and lateral JSWL ≥0.70 ​mm; 101 (12 ​%) had medial JSWL only, and 52 (6 ​%) had lateral JSWL only; 58 (7 ​%) had JSWL ≥0.70 ​mm and pain progression. The majority of participants had follow-up radiographs (and thus JSWL assessed) at 24 months (Table S4). Mean (SD) JSWL was 0.2 ​mm (SD 0.6) and 0.1 ​mm (SD 0.5) in the medial and lateral compartments, respectively. Mean change in the WOMAC knee pain subscore was an improvement of 1.6 points (SD 18, median 1).

**Table 1:** Demographic and clinical characteristics of analytic sample.

Characteristic	n (%) or mean (SD)
Sex	
Female	536 (65 ​%)
Male	291 (35 ​%)
Age (years)	65 (6.6)
BMI (kg/m^2^)	29 (4.8)
KLG	
2	686 (83 ​%)
3	141 (17 ​%)
Baseline WOMAC pain (0–100, 100 worst)	48.0 (15.2)
Baseline medial JSW (mm)	3.7 (1.0)
Baseline lateral JSW (mm)	5.7 (1.3)
**Outcomes**	
Any (Medial or lateral) JWSL ≥0.70 ​mm	159 (19 ​%)
Any (Medial or lateral) JWSL ≥0.50 ​mm	248 (30 ​%)
WOMAC pain progression ≥9 points^a^	212 (26 ​%)
Any (Medial or lateral) JWSL ≥0.70 ​mm AND WOMAC pain progression ≥9 points^a^	58 (7 ​%)
Medial JWSL ≥0.70 ​mm	107 (13 ​%)
Lateral JWSL ≥0.70 ​mm	58 (7 ​%)

a n ​= ​6 missing follow-up pain.

Demographic and clinical characteristics by subgroup (serum biomarkers, urine biomarkers, serum and urine biomarkers) are shown in Supplementary Table S3 and by the study RCT cohort in Table S4. The VIDEO study had a higher percentage of knees with baseline KLG 3, and also a higher percentage of participants with JSWL and pain progression. JSWL was measured through 24 months of follow-up in both oral salmon calcitonin RCTs, while the majority of participants in the VIDEO trial had JSWL measured at 36 months.

Descriptive statistics of the untransformed biomarkers are provided in Supplementary Table S5; correlation between biomarkers is shown in Supplementary Fig. S1. Correlation was high for some biomarkers, for example, a correlation between urinary C2C-HUSA and urinary CTX-II of 0.63, and a correlation between urinary CTX-1β and urinary CTX-II of 0.85. Correlations were low between serum HA and other biomarkers, and between serum PRO-C2 and other biomarkers.

**Fig. 1:**
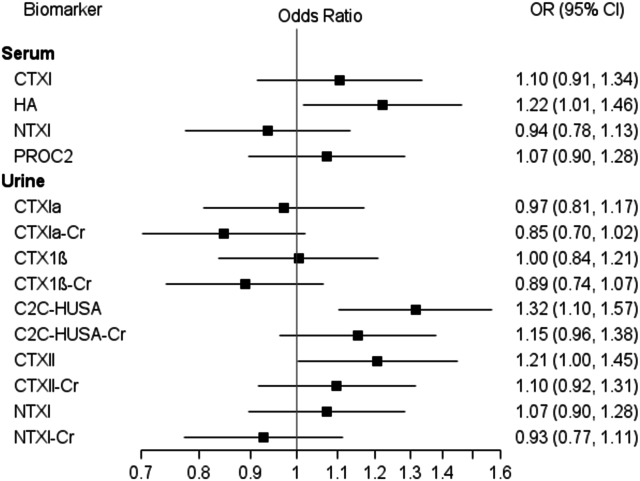
Univariate Associations between biomarkers and primary outcome (JSL ≥0.7 ​mm). Each biomarker is shown along the y-axis and odds ratios (blue square) and associated 95 ​% confidence intervals (horizontal line) are shown along the x-axis. Results are from univariate logistic regression models. Biomarkers were log transformed and standardized prior to analysis; the OR therefore represents the increased odds of JSWL ≥0.7 ​mm for each 1 standard deviation increase in log transformed biomarker. For urine biomarkers “Cr” denotes creatinine normalized.

Univariate associations between each biomarker and the primary outcome of JSWL ≥0.70 ​mm are shown in Fig. 1. Each 1 SD increase in log-transformed sHA (z-score) was associated with a 1.2 times increased odds of JSWL, while a 1 SD increase in log-transformed uC2C-HUSA (z-score) was associated with a 1.3 times increased odds of JSWL. Univariate associations between each biomarker and the secondary outcomes of JSWL ≥0.5 ​mm, knee pain progression, and the composite outcome of JSWL ≥0.7 ​mm and knee pain progression are shown in Figures S2, S3, and S4. The two serum markers of bone resorption, CTXI and NTXI, were both significantly associated with symptomatic progression, with higher concentrations associated with an increased odds of progression: each 1 SD increase in log-transformed sCTXI (z-score) was associated with a 1.2 times increased odds, while a 1 SD increase in NTXI (z-score) was associated with a 1.3 times increased odds of symptomatic progression (Fig. S3).

Results of penalized logistic regression with elastic net for the set of serum biomarkers and baseline covariates are shown in Table 2. HA, BMI, and KLG were selected as the key predictors for JWSL ≥0.70 ​mm, yielding cross-validated AUC 0.608 (95 ​% confidence interval: 0.555, 0.662). The model with only BMI and KLG yielded cross-validated AUC 0.605 (95 ​% CI: 0.552, 0.658) (Table S9). In models without covariates, HA was selected in the model predicting JWSL ≥0.70 ​mm (AUC 0.542, 95 ​% CI: (0.487, 0.596)) (Table S6). HA was also selected in models predicting the secondary outcomes of combined JWSL ≥0.70 ​mm and pain progression, and lateral JWSL ≥0.70 ​mm. No biomarkers were selected by elastic net regression for the prediction of JWSL ≥0.50 ​mm or medial JWSL ≥0.70 ​mm outcomes. In models without covariates, biomarkers selected for other outcomes were generally consistent with the primary analysis with covariates, but overall AUCs were lower.

**Table 2:** Results of penalized logistic regression to predict outcomes from serum biomarkers and covariates (n ​= ​722).

Outcome	Biomarkers				AUC (95 ​% CI)
	sCTX-I	sHA	sNTX-I	sPIIBNP (pro-C2)	
JWSL ≥0.70 ​mm^a^		x			0.608 (0.555, 0.662)
JWSL ≥0.50 ​mm^b^					0.598 (0.552, 0.643)
Pain progression^d^			x		0.549 (0.502, 0.597)
JWSL ≥0.70 ​mm ​+ ​pain progression^b^		x			0.564 (0.479, 0.649)
Medial JWSL ≥0.70 ​mm^c^					0.604 (0.544, 0.663)
Lateral JWSL ≥0.70 ​mm^a^	x	x			0.597 (0.510, 0.685)

Models for each outcome are shown in the rows of the table and biomarkers selected by elastic net for that outcome are shown in the columns. “x” in column indicates that biomarker was selected in >80 ​% of bootstrap samples.

a BMI and KLG selected.

b BMI selected.

c BMI, KLG, sex selected.

d No clinical covariates selecteds.

Results for the set of urine biomarkers, with and without creatine normalization, and baseline covariates are shown in Table 3. For the set of biomarkers without creatinine normalization, C2C-HUSA, BMI, and KLG were selected for predicting JWSL ≥0.70 ​mm yielding AUC 0.617 (95 ​% CI: 0.566, 0.668). The model predicting JWSL ≥0.70 ​mm using covariates only yielded AUC 0.603 (95 ​% CI: 0.554, 0.652) (Table S9). C2C-HUSA was selected by elastic net regression for predicting secondary outcomes, i.e. JWSL ≥0.50 ​mm, medial JWSL ≥0.70 ​mm, and lateral JWSL ≥0.70 ​mm (Table 3). Using creatinine normalization, fewer biomarkers were identified as predictors and had similar or lower AUCs compared to the models without creatinine normalization (Table 3). No biomarkers were selected in elastic net models using creatinine normalized biomarkers to predict the outcomes of JSWL ≥0.70 ​mm, JSWL ≥0.50 ​mm, medial JSWL ≥0.70 ​mm, or lateral JSWL ≥0.70 ​mm. In models that did not include covariates, with the exception of pain progression, C2C-HUSA was selected for all outcomes (Table S7). The AUC for the model predicting JSWL ≥0.70 ​mm from C2C-HUSA alone was 0.568 (95 ​% CI: 0.518, 0.618).

**Table 3:** Results of penalized logistic regression to predict outcomes from urine biomarkers and covariates (n ​= ​786).

Outcome	Biomarkers					AUC (95 ​% CI)
	uCTXIα	uCTXIβ	uC2C-HUSA	uCTXII	uNTXI	
*Non-creatinine normalized*						
JWSL ≥0.70 ​mm^a^			x			0.617 (0.566, 0.668)
JWSL ≥0.50 ​mm^b^			x			0.614 (0.571, 0.657)
Pain progression^e^						–
JWSL ≥0.70 ​mm ​+ ​pain progression^a^						0.550 (0.469, 0.630)
Medial JWSL ≥0.70 ​mm^a^			x			0.603 (0.545, 0.660)
Lateral JWSL ≥0.70 ​mm^c^			x			0.601 (0.509, 0.693)
*Creatinine normalized.*						
JWSL ≥0.70 ​mm^a^						0.609 (0.558, 0.660)
JWSL ≥0.50 ​mm^b^						0.608 (0.565, 0.651)
Pain progression^e^						–
JWSL ≥0.70 ​mm ​+ ​pain progression^e^			x			0.576 (0.489, 0.663)
Medial JWSL ≥0.70 ​mm^d^						0.606 (0.548, 0.663)
Lateral JWSL ≥0.70 ​mm^c^						0.585 (0.497, 0.674)

“x” in column indicates that biomarker was selected in >80 ​% of bootstrap samples.

a BMI, KLG selected.

b BMI selected.

c Age, BMI, selected.

d BMI, KLG, Sex selected.

e No covariates or biomarkers selected >80 ​% of bootstrap samples.

Table 4 shows models including both serum and urine biomarkers, with and without creatinine normalization. For the set of models with creatinine normalized urine biomarkers, the combination of serum HA, urine C2C-HUSA, BMI, and KLG was selected to predict the primary outcome of JWSL ≥0.70 ​mm, yielding AUC 0.627 (95 ​% CI: 0.573, 0.681). The model predicting JWSL ≥0.70 ​mm using covariates only yielded AUC 0.612 (95 ​% CI: 0.557, 0.667) (Table S9).

**Table 4:** Results of penalized logistic regression to predict outcomes from urine biomarkers and covariates (n ​= ​681).

Outcome	Serum				Urine					AUC (95 ​% CI)
	CTX-I	HA	NTX-I	PIIBNP (pro-C2)	CTXIα	CTXIβ	C2C-HUSA	CTXII	NTXI	
*Non-creatinine normalized*										
JWSL ≥0.70 ​mm^a^		x					x			0.627 (0.573, 0.681)
JWSL ≥0.50 ​mm^b^			x				x			0.622 (0.576, 0.668)
Pain progression			x							0.558 (0.509, 0.608)
JWSL ≥0.70 ​mm ​+ ​pain progression^c^		x		x						0.588 (0.496, 0.680)
Medial JWSL ≥0.70 ​mm^d^		x					x			0.621 (0.560, 0.682)
Lateral JWSL ≥0.70 ​mm^b^		x					x			0.604 (0.506, 0.680)
*Creatinine normalized*										
JWSL ≥0.70 ​mm^a^		x								0.626 (0.572, 0.681)
JWSL ≥0.50 ​mm^b^										0.607 (0.560, 0.654)
Pain progression			x							0.558 (0.509, 0.608)
JWSL ≥0.70 ​mm ​+ ​pain progression^e^		x	x	x			x	x	x	0.602 (0.517, 0.686)
Medial JWSL ≥0.70 ​mm^d^		x								0.625 (0.563, 0.686)
Lateral JWSL ≥0.70 ​mm^b^		x								0.602 (0.507, 0.698)

“x” in column indicates that biomarker was selected in >80 ​% of bootstrap samples.

a BMI, KLG selected.

b BMI selected.

c Age, BMI, selected.

d BMI, KLG, sex selected.

e Age, BMI, sex selected.

Urine C2C-HUSA was selected as a predictor for all outcomes with the exception of pain progression and the composite outcome of JWSL ≥0.70 ​mm and pain progression. Serum HA was selected in models for all outcomes with the exception of pain progression and JSWL ≥0.50 ​mm. Results from biomarkers, singly or in combination, without covariates, were generally comparable with AUCs ≤0.60 (Table S8). Serum HA, serum NTX-I, and urine C2C-HUSA were consistently selected by the elastic net models. The combination of serum HA and urine C2C-HUSA was selected to predict the primary outcome of JSWL ≥0.70 ​mm, yielding AUC 0.581 (95 ​% CI: 0.526, 0.636).

Models predicting outcomes using covariates only are shown in Table S9. BMI was selected for most models. AUCs for the pain progression outcome were under 0.5 for all three analyses (i.e., the subgroup with serum biomarkers, urine biomarkers, or both).

Optimal thresholds applied to predict JSL ≥0.7 ​mm are shown in Table 5. AUCs ranged from 0.489 (uBeta CTX-I) to 0.583 (uC2C-HUSA). None of the biomarkers demonstrated a Youden J Statistic of >50.

**Table 5:** Optimal cut-points to predict JSL ≥0.70 ​mm.

Biomarker	Units	Optimal cut-point	Sensitivity	Specificity	AUC
sCTX-I	ng/mL	0.241	45.5 ​%	62.0 ​%	0.495
sHA	ng/mL	46.3	65.9 ​%	48.4 ​%	0.577
sNTX-I	nM BCE	22.9	83.7 ​%	22.2 ​%	0.519
sPIIBNP (pro-C2)	ng/mL	25.68	31.7 ​%	76.3 ​%	0.521
uCTXIα	ng/mL	3.87	36.6 ​%	70.3 ​%	0.503
uCTXIα/CR	μg/mmol	0.6	70.7 ​%	38.7 ​%	0.545
uCTXIβ	ng/mL	10.95	54.5 ​%	55.9 ​%	0.531
uCTXIβ/CR	μg/mmol	4.31	8.1 ​%	93.4 ​%	0.489
uC2C-HUSA	pg/mL	729	72.4 ​%	44.4 ​%	0.583
uC2C-HUSA/CR	ng/mmol	158	47.2 ​%	61.6 ​%	0.543
uCTXII	μg/L	1.11	75.6 ​%	37.6 ​%	0.559
uCTXII/CR	μg/mmol	240	63.4 ​%	48.0 ​%	0.546
uNTXI	nM BCE	313	56.1 ​%	56.8 ​%	0.539
uNTXI/CR	nM BCE/mmol	33	21.1 ​%	85.3 ​%	0.511

CR= creatinine normalized.

## 4 Discussion

In Phase II of the FNIH OA Biomarkers Consortium, the PROGRESS OA study, we sought to externally validate nine biochemical biomarkers previously found to have modest prognostic ability for clinically relevant knee OA progression outcomes related to both radiographic structure and pain symptoms [15,16]. Serum HA and urinary C2C-HUSA were consistently selected in elastic net models across the various analyses and outcomes. The AUC for predicting radiographic progression was 0.627 in the model for the primary outcome of JWSL ≥0.70 ​mm, including serum (HA) and urinary (C2C-HUSA, CTX-II) biomarkers, along with covariates.

Univariate associations were generally similar in direction and magnitude compared with predictive models described in our Phase I study (Fig. 1; Kraus et al. Table 4) [15]. Of particular interest, higher levels of serum HA and urinary C2C-HUSA were associated with an increased risk of radiographic progression. In Phase I, baseline concentrations of both markers were associated with increased risk of progression in all three case groups (JSWL only progression, pain only progression, JSWL and pain progression). In elastic net models including both serum and urine biomarkers in PROGRESS OA, both serum HA and urinary C2C-HUSA were selected for the radiographic progression outcomes of JSWL≥0.7 ​mm (any medial or lateral JSWL, medial JSWL, or lateral JSWL). These two biomarkers had the highest AUCs in models where a threshold was applied; a threshold of 729 ​pg/mL in urinary C2C-HUSA yielded AUC 0.583 and a threshold of 46.3 ​ng/mL in serum HA yielding AUC 0.577 to predict medial or lateral JSWL ≥0.7 ​mm.

In Phase I, multivariable models of baseline biomarkers predicted the combined outcome of medial JSWL ≥0.7 ​mm and WOMAC pain with AUCs ranging between 0.563 and 0.588 (Kraus et al., Table S8, AUCs not cross-validated) [15]. In PROGRESS OA, we found similar or slightly improved model performance when predicting the combined outcome of medial or lateral JSWL ≥0.7 ​mm and WOMAC pain, with cross-validated AUCs ranging from 0.564 (serum biomarkers, Table 2) to 0.602 (urine and serum biomarkers, Table 4).

The inclusion of biochemical biomarkers generally improved model discrimination from models with covariates only. For example, in the analysis limited to participants with urine biomarkers, the AUC for predicting JSWL ≥0.70 ​mm was 0.603 using BMI alone, improving to 0.617 with the addition of urinary C2C-HUSA. However, there were a number of outcomes for which no biomarkers met the >80 ​% threshold elastic net selection criterion for inclusion, in particular, the urinary markers with creatinine normalization (Table 3).

The primary outcome used in our previous Phase I nested case-control study was combined medial JSWL ≥0.7 ​mm and knee pain progression. PROGRESS OA (Phase II) used real world data from the control arms of clinical trials, thus the interpretation of longitudinal pain posed challenges due to placebo and contextual effects and regression to the mean [25]. For this reason, we chose radiographic progression as the primary outcome. Another difference is that PROGRESS OA utilized data on both the medial and lateral compartments (where available), allowing for the assessment of JSWL across multiple compartments. Serum and urine biomarkers are inherently systemic rather than localized to a specific joint or joint compartment and thus reflect the OA burden across the entire body [26]. A non-trivial number of participants (52, 6 ​%) experienced lateral JSWL. In nearly all analyses, we observed higher AUCs in models predicting the combined medial and lateral JSWL outcome. This supports our hypothesis that these systemic biomarkers are better predictors when a more comprehensive accounting is taken of the OA burden. A limitation of this study is the lack of data on OA status in the contralateral knee and other joint sites. Future studies of serum and urine biomarkers in OA should ideally account for how OA in other joints influences systemic biomarker levels, including evaluating the contribution of individual joints to biomarker levels and assessing the biomarkers' ability to predict progression in a specific joint.

The prognostic capacity of this set of serum and urine biochemical biomarkers for predicting clinically relevant progression was weak to modest in both Phase I and PROGRESS OA. AUCs for multivariable models of baseline biomarkers did not surpass 0.6 in Phase I for predicting combined medial JSWL ≥0.7 ​mm and pain progression, and did not surpass 0.63 to predict JSWL ≥0.7 ​mm in PROGRESS OA. These findings suggest that this particular panel of biomarkers may have limited utility for prognostic enrichment, highlighting the need to explore emerging biomarkers with stronger prognostic potential. In a follow-up analysis of the Phase I sample, Zhou and colleagues quantitatively measured 177 peptides and then used elastic net selection to find the best combination of biomarkers to predict the Phase I primary outcome of combined medial JSWL ≥0.7 ​mm and WOMAC pain progression. The final model included 15 serum proteomic markers corresponding to 13 total proteins yielding AUC 0.729 using biomarkers alone and AUC 0.743 using the combination of these markers and covariates. Adding urinary CTX-II increased the AUC to 0.771 [27]. A panel of 11 of these serum proteomic biomarkers was validated in an independent cohort, with an AUC of 0.72 [28]. This represents a promising future direction for prognostic biochemical biomarkers in knee OA, especially since serum is relatively easy and inexpensive to obtain.

The goal in identifying prognostic biomarkers and in developing prognostic enrichment algorithms is to inform clinical trial design and recruitment strategies – ‘can we enroll patients at a high risk of trial outcomes, and if so, what are the implications for trial size and cost?’ Kerr et al. describe several factors that must be considered to determine whether it is worthwhile to deploy a prognostic enrichment strategy [12]. These authors developed an R package, the Biomarker Prognostic Enrichment Tool, to weigh these factors and inform decisions regarding when or whether to incorporate an enrichment strategy [12]. These authors show that even a biomarker with modest discrimination can result in cost savings for a trial. In their example, a biomarker with an AUC of 0.63, which is similar to our best performing models, can result in cost savings if the cost of the screening is low; in this example, the cost of screening is US$50 and the per-patient trial cost is US$1000. In addition to the cost of deploying a prognostic enrichment algorithm, i.e., the cost of the additional screening, and the per-patient trial cost, we must also consider the accuracy of the enrichment algorithm, the anticipated outcome rate in an unenriched cohort, the anticipated treatment efficacy, as well as less quantitative considerations such as the generalizability of the enriched cohort, which may affect regulatory decisions regarding drug labeling and approved indications [12,13]. In this study, we aimed to externally validate biomarkers for prognostic prediction of knee OA progression. Future work investigating specific clinical trial settings will be needed to evaluate the feasibility and utility of implementing these biomarkers as enrichment tools.

Here, we investigated serum and urine biomarkers as prognostic markers, that is, the ability to predict “the progression of OA among those with existing disease” [29]. Efficacy of intervention biomarkers, on the other hand, “may be measured prior to therapy to predict treatment efficacy, or may be measured more than once to assess short-term changes that occur as a result of pharmacologic or other interventions” with subset categories that include monitoring biomarkers, response biomarkers, predictive biomarkers, and surrogate end points [9,30]. A recent RCT used CTX-I, a biomarker of bone resorption, and urine CTX-II, a biomarker of cartilage loss, to evaluate treatment efficacy [31]. The trial evaluated the effect of MIV-711, a novel selective cathepsin K inhibitor, on pain and structural progression in participants with knee OA. Significant differences were observed between intervention and placebo groups when assessing change in biochemical markers from baseline to 26-weeks follow-up. Another recent study investigated PRO-C2, a serum marker of type II collagen formation, as a predictive biomarker in the FORWARD study, an RCT testing the efficacy of intra-articular sprifermin [32,33]. A secondary analysis found that participants with low levels of PRO-C2 at baseline had better response to treatment – increased cartilage thickness over time and decreased pain – compared to those with high levels of PRO-C2 [33]. These studies suggest that indeed such biomarkers may be candidates for efficacy of intervention markers. Future work includes utilizing the rich PROGRESS OA cohort to investigate short-term changes in biochemical biomarkers to assess whether they have potential as ‘monitoring biomarkers’; that is, biomarkers that reflect change in disease status, or that could act as surrogate endpoints to predict clinical benefit or harm.

This study should be interpreted in the context of several limitations. As noted above, the interpretation of pain changes in the placebo arm of an RCT is challenging as improvements in pain may reflect regression to the mean or placebo effects [25,34]. On average, there was little change in WOMAC pain across the three RCTs covering a period of up to 36 months of follow-up. Biochemical biomarker data were not available for all participants for all trials, and in some trials and/or study sites, both serum and urine markers were not simultaneously collected. Only a limited subset of demographic and clinical factors was made available, and so we decided not to undertake imputation for missing data as there was insufficient information on which to base imputation. The vast majority of participants came from just two of the three contributing RCTs. The initial goal of this study was to investigate the combination of biochemical and imaging biomarkers to predict clinically relevant progression, as explored in Phase I. However, only one trial (VIDEO) included both biochemical and imaging biomarkers, but its sample size was too small to support this analysis [16,35]. While these biochemical biomarkers demonstrated modest discrimination in predicting progression in both the Phase I and PROGRESS OA studies, a key step in deploying these in a clinical trial context is to demonstrate whether such an algorithm facilitates the selection of patients at high risk of progression, and whether such selection meaningfully impacts trial effect sizes.

In conclusion, we sought to externally validate a set of prognostic biochemical biomarkers for clinically relevant knee OA progression outcomes that encompassed radiographic and/or pain-related changes. Serum HA and urinary C2C-HUSA were consistently selected as important predictors across the various analytic datasets and outcomes. While associations and prognostic performance were generally similar to our previous Phase I study, discrimination was modest in both studies. Additional biochemical biomarkers in development, such as those identified through serum proteomic analyses, may offer better discrimination in identifying OA progression and therefore represent a path forward for a prognostic and predictive contexts of use [27].

## Author contributions

Substantial contributions to study conception and design – JEC, LAD, CJS, DJH, VBK.

Substantial contributions to acquisition of data – DJH, VBK.

Substantial contributions to analysis and interpretation of data – all authors.

Drafting the article or revising it critically for important intellectual content – all authors.

Final approval of the version of the article to be published – all authors.

JEC takes responsibility for the integrity of the work as a whole, from inception to finished article.

## Role of the funding source

Scientific and financial support for the FNIH Biomarkers Consortium PROGRESS OA study is made possible through grants and direct and in-kind contributions provided by the following: Arthritis Foundation; Biosplice Therapeutics Inc.; Merck KGaA, Darmstadt, Germany; Novartis; Pfizer.

Study sponsors were not involved in the study design, analysis and interpretation of data; in the writing of the manuscript; or in the decision to submit the manuscript for publication.

## Declaration of competing interest

JEC has received consulting fees from Boston Imaging Core Labs, LLC.

DJH is the editor of the osteoarthritis section for UpToDate and co-Editor in Chief of Osteoarthritis and Cartilage. DJH provides consulting advice on scientific advisory boards for Haleon, TLCBio, Novartis, Tissuegene, Sanofi, Enlivex.

VBK received a grant from the Foundations for the National Institutes of Health (to her institution) related to this work and consulting fees unrelated to this work from 4-P Pharma, Novartis and Paradigm Biopharma.

CL is a self-employed owner of CHL4special consulting. He has provided consulting services to Regenosine, Curnova, Charité hospital, TrialSpark/Formation Bio, Gordian and ReumaNederland. He was formerly an employee of Merck KGaA.

LAD receives royalties from UptoDate.
