# Supplementary material for: Biochemical biomarkers of knee osteoarthritis progression: Results from the FNIH biomarkers consortium progress OA study
Source: Osteoarthr Cartil Open. 2025 Sep 5;7(4):100677. doi: 10.1016/j.ocarto.2025.100677 (PMC12495463; doi:10.1016/j.ocarto.2025.100677)
Supplement: Multimedia component 1 [file mmc1.docx]

**Supplementary Materials**

**Biochemical Biomarkers of Knee Osteoarthritis Progression: Results from The FNIH Biomarkers Consortium Progress OA Study**

Contents

[Table S1. Biochemical markers assessed in PROGRESS OA Study 2](#_Toc180496986)

[Table S2. Biochemical Biomarkers Technical Performance 4](#_Toc180496987)

[Table S3. Demographic and Clinical Characteristics by Analysis Cohort. 6](#_Toc180496988)

[Table S4. Demographic and Clinical Characteristics by Study. 7](#_Toc180496989)

[Figure S1. Pearson Correlation between biomarkers 9](#_Toc180496990)

[Figure S2. Univariate Associations between biomarkers and secondary outcome JSL ≥ 0.5mm 10](#_Toc180496991)

[Figure S3. Univariate Associations between biomarkers and secondary outcome WOMAC Pain Progression 11](#_Toc180496992)

[Figure S4. Univariate Associations between biomarkers and secondary outcome JSWL ≥ 0.7mm and WOMAC Pain Progression 12](#_Toc180496993)

[Table S6. Results of penalized logistic regression to predict outcomes from serum biomarkers (covariates not included) (n=722). 13](#_Toc180496994)

[Table S7. Results of penalized logistic regression to predict outcomes from urine biomarkers (covariates not included) (n=786). 14](#_Toc180496995)

[Table S8. Results of penalized logistic regression to predict outcomes from serum and urine biomarkers (covariates not included) (n=681). 15](#_Toc180496996)

[Table S9. Cross-Validated AUCs for models with covariates only. 16](#_Toc180496997)

# Table S1. Biochemical markers assessed in PROGRESS OA Study

| **Biomarker** | **Full Name** | **Manufacturer (Cat. Number)** | **Rationale** | **Results from Phase 1** |
| --- | --- | --- | --- | --- |
| uCTXII | C-terminal crosslinked telopeptide type II collagen | IDS (AC-10F1) | Multivariable modeling results | Associated with all three progressor groups; odds of predicting case status were stronger comparing cases to 'pure' non-progressors; baseline, 12M and 24M time-integrated concentration (TIC) predict primary status; every timepoint (baseline, 12M and 24M) predicts every progressor type in secondary analyses |
| sHA | hyaluronan | Corgenix (029-001) | Multivariable modeling results | Stronger odds of predicting case status when cases were compared to 'pure' non-progressors; 24M TIC predicts case status in primary analysis; 12M and 24M TIC predict case status in secondary analysis |
| sNTXI | N-telopeptide of type I collagen | ALERE -Osteomark (Inverness Medical) (9021) | Multivariable modeling results | 12M and 24M TIC predict case status in primary and secondary analysis |
| uC2C-HUSA | C-terminal cleavage product of human type II collagen human urinary sandwich assay | IBEX (60-1017) | Univariable modeling results (unadjusted and adjusted) | Associated with all three progressor groups; odds of predicting case status were stronger comparing cases to 'pure' non-progressors; 24M TIC predicts primary status; every timepoint (except baseline for structural outcome) predicts every progressor type in secondary analyses |
| sCTXI | C-terminal crosslinked telopeptide of type I collagen | IDS (AC-02F1) | Univariable modeling results (unadjusted and adjusted) | Stronger odds of predicting case status when cases were compared to 'pure' non-progressors; 12M and 24M TIC predict case status in primary analysis; all timepoints predict case status in secondary analyses |
| sPIIBNP (pro-C2) | N-terminal propeptide of type IIB collagen | Nordic Bioscience (Herlev, Denmark) | Univariable modeling results (unadjusted and adjusted) | In two longitudinal cohorts (NYU and SMC), study participants with low PRO-C2 levels had greater joint space loss compared with subjects with high PRO-C2 corresponding to a 3.4 higher risk of knee OA progression |
| uCTXIα | Alpha isomerized versions of CTXI | IDS (AC-04F1) | Univariable modeling results (unadjusted and adjusted) | Stronger odds of predicting case status when cases were compared to 'pure' non-progressors; baseline, 12M and 24M TIC predict case status in primary analysis; 12M and 24M TIC predict case status in secondary analyses |
| uCTXIβ | beta isomerized version of CTXI | IDS (AC-05F1) | Univariable modeling results (unadjusted and adjusted) | Stronger odds of predicting case status when cases were compared to 'pure' non-progressors; 12M and 24M TIC predict case status in primary and secondary analyses |
| uNTXI | N-telopeptide of type I collagen | ALERE -Osteomark (Inverness Medical) (9006) | Univariable modeling results (unadjusted and adjusted) | Stronger odds of predicting case status when cases were compared to 'pure' non-progressors; 12M and 24M TIC predict case status in primary and secondary analyses |
| uCreatinine | Creatinine (for normalization of urinary markers) | Quidel MicroVue (8009) | To normalize urinary markers |  |

# Table S2. Biochemical Biomarkers Technical Performance

| **Matrix** | **Biomarker** | **Abbrev** | **Assay type** | **Intra-assay CV (%)** | **Inter-assay CV (%)** | **ULOQ (ng/ml) (n>ULOQ)** | **LLOQ (ng/ml) (n<LLOQ))** | **Missing (n)** | **Normal range (ng/ml)** | **Sample dilution** |
| --- | --- | --- | --- | --- | --- | --- | --- | --- | --- | --- |
| Serum | Hyaluronan | HA | Sandwich ELISA | < 7.2% | < 16.2% | 800.0 (0) | 20.0 (39) | 3 | 20.0 – 195.5 | 1:11 |
|  | N-Terminal pro-peptides of type II collagen | Pro-C2 | Competitive CLIA | < 6.6% | < 12.4% | 789.5 (0) | 10.3 (195) | 4 | 10.3 – 21.2 | undiluted |
|  | Cross-linked N-telopeptides of type I collagen | NTX | Competitive-Inhibition ELISA | < 9.8% | < 19.5% (n=182 > 20%) | 32.0 (45) | 4.0 (2) | 2 | 6.2 – 19.0 (women); 5.4 – 24.2 (men) | 1:4 |
|  | Neo-epitopes of cathepsin mediated degradation of type I collagen (ß-Crosslaps) | CTX-I | Sandwich ECLIA | < 8.9% | < 5.0% | 6.0 (0) | 0.010 (0) | 2 | 0.104 – 1.008 (postmeno women); 0.010 – 0.704 (men 50-70 years) | undiluted |
| Urine | Degradation products of C-terminal telopeptides of Type II collagen in human urine | CTX-II | Competitive ELISA | < 8.5% | < 6.4% | 10.73 (0) | 0.29 (21) | 12 | 65-618 (premeno women); 112-1172 (postmeno women); 87-895 (men) | undiluted |
|  | Cross-linked N-telopeptides of type I collagen) | NTX | Competitive-Inhibition ELISA | < 4.7% | < 9.3% | 2500 (0) | 40 (1) | 15 | 5-65 (postmeno women); 3-63 (men) | undiluted |
|  | Degradation products of C-terminal telopeptides of collagen type I) | CTX-I𝛼 | ELISA | < 7.14% | < 15.18% | 47.84 (0) | 0.20 (232) | 12 | 0.10 – 0.99 (premeno women); 0.17 – 2.26 (postmeno women)’ 0.13 – 1.13 (men) | 1:8 |
|  | Degradation products of C-terminal telopeptides of collagen type I) | CTX-Iß | ELISA | < 6.4% | < 10.8% | 79.0 (0) | 0.80 (3) | 13 | 0.83 – 3.32 (premeno women); 0.73 – 7.07 (postmeno women); 0.53 – 4.04 (men) | 1:4 |
|  | Neoepitope created at C-terminus of the 3/4 peptide of type II collagen generated through cleavage by collagenases) | C2C-HUSA | Antigen Sandwich ELISA | < 2.7% | < 5.6% | 10000 (0) | 403 (161) | 12 | ND | 1:2 |
|  | Creatinine | Cr | Enzymatic | < 0.6% | < 1.3% | 21.7 | 0.09 | 7 | NA | undiluted |

698 human serum samples; 806 human urine samples

ULOQ upper limit of quantification; LLOQ lower limit of quantification

Missingness: sample missing, sample empty, incorrect type of sample, unacceptable CV in 3 runs)

# Table S3. Demographic and Clinical Characteristics by Analysis Cohort.

Shown in cells: n (%) or mean (SD).

| **Characteristic** | **Serum (n=722)** | **Urine (n=786)** | **Serum + Urine (n=681)** |
| --- | --- | --- | --- |
| Sex |  |  |  |
| Female | 528 (73%) | 507 (65%) | 499 (73%) |
| Male | 194 (27%) | 279 (35%) | 182 (27%) |
| Age (years) | 64.7 (6.5) | 64.5 (6.5) | 64.7 (6.4) |
| BMI (kg/m^2^) | 28.6 (4.9) | 28.6 (4.8) | 28.6 (4.8) |
| KLG |  |  |  |
| 2 | 595 (82%) | 657 (84%) | 566 (83%) |
| 3 | 127 (18%) | 129 (16%) | 115 (17%) |
| Baseline WOMAC pain (0 -100, 100 worst) | 48.3 (15.6) | 48.5 (14.5) | 48.9 (14.9) |
| Baseline medial JSW (mm) | 3.6 (1.0) | 3.6 (1.0) | 3.6 (1.0) |
| Baseline lateral JSW (mm) | 5.6 (1.3) | 5.7 (1.3) | 5.6 (1.3) |
| **Outcomes** |  |  |  |
| Any (Medial or Lateral) JSL ≥ 0.70mm | 139 (19%) | 143 (18%) | 123 (18%) |
| Any (Medial or Lateral) JSL ≥ 0.50mm | 215 (30%) | 231 (29%) | 198 (29%) |
| WOMAC pain progression ≥9 points* | 189 (26%) | 192 (25%) | 169 (25%) |
| Any (Medial or Lateral) JSL ≥ 0.70mm  AND WOMAC pain progression ≥9 points* | 52 (7%) | 49 (6%) | 43 (6%) |
| Any (Medial or Lateral) JSL ≥ 0.50mm  AND WOMAC pain progression ≥9 points* | 70 (10%) | 69 (9%) | 61 (9%) |

# Table S4. Demographic and Clinical Characteristics by Study.

Shown in cells: n (%) or mean (SD).

| **Characteristic** | **Novartis 2301**  **(NCT00486434)** | **Novartis 2302**  **(NCT00704847)** | **VIDEO**  **(ISRCTN 94818153)** |
| --- | --- | --- | --- |
| Sex |  |  |  |
| Female | 311 (64%) | 202 (65%) | 23 (72%) |
| Male | 172 (36%) | 110 (35%) | 9 (28%) |
| Age (years) | 64.5 (6.4) | 64.6 (6.7) | 64.0 (7.6) |
| BMI (kg/m^2^) | 28.6 (4.5) | 28.7 (5.1) | 29.3 (5.9) |
| KLG |  |  |  |
| 2 | 422 (87%) | 243 (78%) | 21 (66%) |
| 3 | 61 (13%) | 69 (22%) | 11 (34%) |
| Baseline WOMAC pain (0 -100, 100 worst) | 47.8 (14.9) | 50.0 (14.2) | 32.4 (20.2) |
| Baseline medial JSW (mm) | 3.6 (1.0) | 3.7 (1.0) | 4.0 (1.2) |
| Baseline lateral JSW (mm) | 5.6 (1.3) | 5.7 (1.2) | 5.5 (2.0) |
| **Outcomes** |  |  |  |
| Any (Medial or Lateral) JWSL ≥ 0.70mm | 81 (17%) | 63 (20%) | 15 (47%) |
| Any (Medial or Lateral) JWSL ≥ 0.50mm | 136 (28%) | 96 (31%) | 16 (50%) |
| WOMAC pain progression ≥9 points* | 117 (24%) | 76 (25%) | 19 (59%) |
| Any (Medial or Lateral) JWSL ≥ 0.70mm  AND WOMAC pain progression ≥9 points* | 21 (4%) | 14 (5%) | 6 (19%) |
| Medial JWSL ≥ 0.70mm | 57 (12%) | 40 (13%) | 10 (31%) |
| Lateral JWSL ≥ 0.70mm | 27 (6%) | 24 (8%) | 7 (22%) |
| **Biomarkers** |  |  |  |
| Serum biomarkers available | 467 (97%) | 223 (71%) | 32 (100%) |
| Urine biomarkers available | 475 (98%) | 311 (100%) | 0 (0%) |
| Serum and Urine biomarkers available | 459 (95%) | 222 (71%) | 0 (0%) |
| **Month of JSW Follow-up** |  |  |  |
| 12 | 0 (0%) | 0 (0%) | 6 (19%) |
| 24 | 483 (100%) | 312 (100%) | 3 (9%) |
| 36 | 0 (0%) | 0 (0%) | 23 (72%) |

*n=6 missing follow-up pain

Table S5. Descriptive Statistics of Biochemical Biomarkers

| **Variable** | **N** | **Mean** | **Median** | **Std Dev** | **Min** | **Max** |
| --- | --- | --- | --- | --- | --- | --- |
| s-CTX-I | 722 | 0.24 | 0.11 | 0.22 | 0.04 | 0.75 |
| s-HA | 722 | 62.35 | 51.06 | 49.50 | 20.00 | 553.40 |
| s-NTX-I | 722 | 18.59 | 5.48 | 18.20 | 5.20 | 38.50 |
| s-PRO-C2 | 722 | 22.06 | 11.91 | 20.12 | 10.20 | 151.98 |
| u-αCTX-I | 786 | 3.52 | 2.42 | 2.73 | 1.60 | 25.21 |
| u-αCTX-I/CR | 786 | 0.60 | 0.45 | 0.49 | 0.11 | 5.89 |
| u-βCTX-I | 786 | 13.12 | 10.24 | 10.35 | 0.80 | 111.30 |
| u-βCTX-I/CR | 786 | 2.11 | 1.48 | 1.76 | 0.15 | 19.88 |
| u-C2C-HUSA | 786 | 955.25 | 557.84 | 782.50 | 403.00 | 4362.00 |
| u-C2C-HUSA/CR | 786 | 156.21 | 99.92 | 139.00 | 51.00 | 2182.00 |
| u-CTX-II | 786 | 6.70 | 3.38 | 5.89 | 0.83 | 20.00 |
| u-CTX-II/CR | 786 | 1.88 | 1.68 | 1.44 | 0.20 | 19.64 |
| u-NTX-I | 786 | 298.34 | 221.75 | 246.50 | 22.00 | 1938.00 |
| u-NTX-I/CR | 786 | 361.39 | 254.57 | 293.50 | 40.00 | 3281.00 |

*s=serum; u=urine; CR=* *creatinine normalized.*

# Figure S1. Pearson Correlation between biomarkers


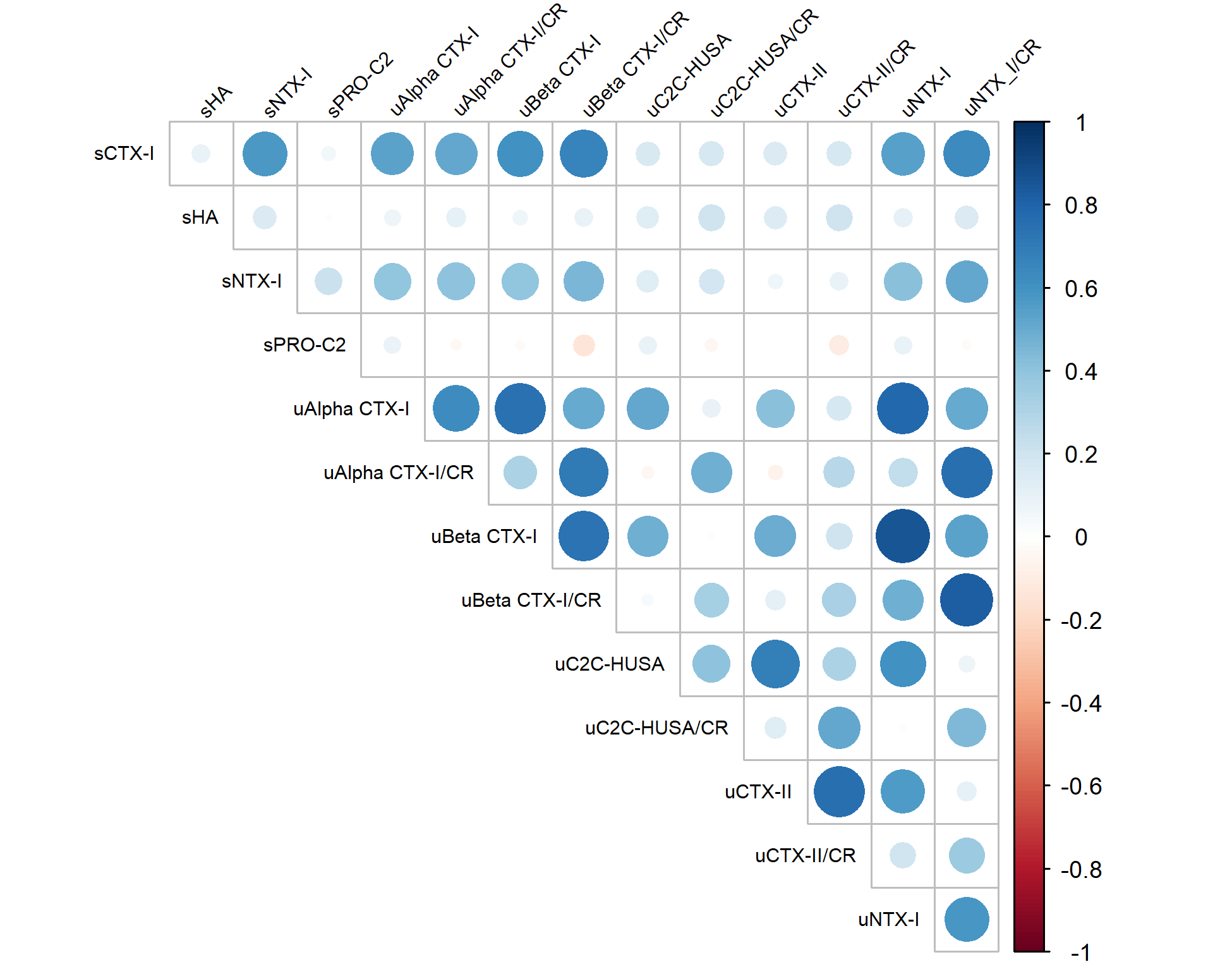


*CR=* *creatinine normalized.*

# Figure S2. Univariate Associations between biomarkers and secondary outcome JSL ≥ 0.5mm


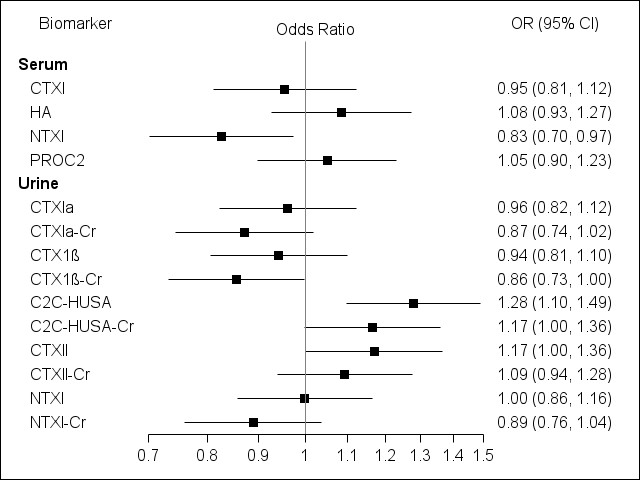


*Biomarkers log-transformed and standardized. Cr=* *creatinine normalized.*

# Figure S3. Univariate Associations between biomarkers and secondary outcome WOMAC Pain Progression

*
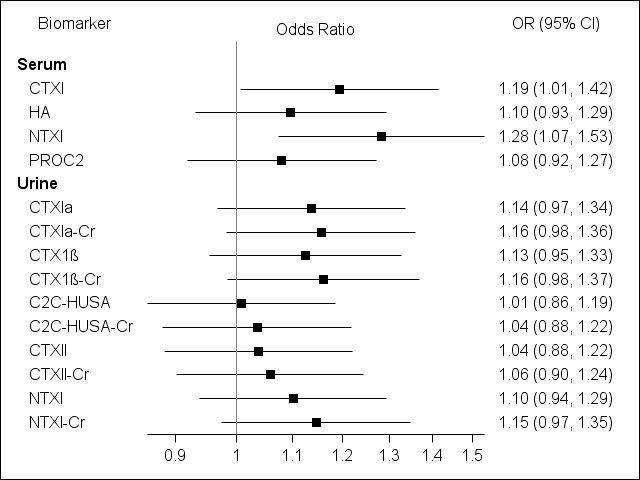
*

*Biomarkers log-transformed and standardized. Cr=* *creatinine normalized.*

# Figure S4. Univariate Associations between biomarkers and secondary outcome JSWL ≥ 0.7mm and WOMAC Pain Progression


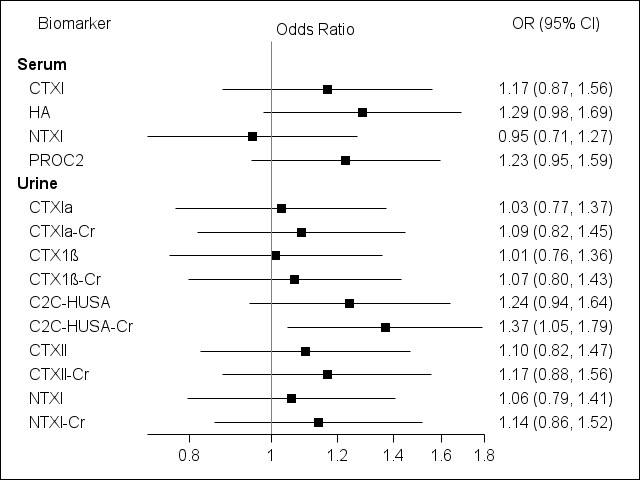


*Biomarkers log-transformed and standardized. Cr=* *creatinine normalized.*

# Table S6. Results of penalized logistic regression to predict outcomes from serum biomarkers (covariates not included) (n=722).

| **Outcome** | **Biomarkers** | | | | **AUC** |
| --- | --- | --- | --- | --- | --- |
|  | **sCTX-I** | **sHA** | **sNTX-I** | **sPIIBNP (pro-C2)** |  |
| JWSL ≥ 0.70mm |  | x |  |  | 0.542 (0.487, 0.596) |
| JWSL ≥ 0.50mm |  |  | x |  | 0.532 (0.486, 0.577) |
| Pain progression |  |  | x |  | 0.549 (0.502, 0.597) |
| JWSL ≥ 0.70mm + pain progression | x | x |  | x | 0.522 (0.437, 0.606) |
| Medial JWSL ≥ 0.70mm |  | x | x |  | 0.525 (0.462, 0.587) |
| Lateral JWSL ≥ 0.70mm | x | x |  |  | 0.533 (0.445, 0.621) |

# Table S7. Results of penalized logistic regression to predict outcomes from urine biomarkers (covariates not included) (n=786).

| **Outcome** | **Biomarkers** | | | | | **AUC** |
| --- | --- | --- | --- | --- | --- | --- |
|  | **uCTXIα** | **uCTXIβ** | **uC2C-HUSA** | **uCTXII** | **uNTXI** |  |
| *Non-creatinine normalized* | | | | | | |
| JWSL ≥ 0.70mm |  |  | x |  |  | 0.568 (0.518, 0.618) |
| JWSL ≥ 0.50mm |  |  | x |  |  | 0.564 (0.520, 0.607) |
| Pain progression | x |  |  |  |  | 0.502 (0.455, 0.550) |
| JWSL ≥ 0.70mm + pain progression |  |  | x |  |  | 0.497 (0.413, 0.581) |
| Medial JWSL ≥ 0.70mm |  |  | x |  |  | 0.561 (0.503, 0.620) |
| Lateral JWSL ≥ 0.70mm |  |  | x |  |  | 0.563 (0.481, 0.644) |
| *Creatinine normalized.* | | | | | | |
| JWSL ≥ 0.70mm | x |  | x |  |  | 0.564 (0.511, 0.617) |
| JWSL ≥ 0.50mm | x | x | x |  |  | 0.563 (0.520, 0.607) |
| Pain progression* |  |  |  |  |  | -- |
| JWSL ≥ 0.70mm + pain progression |  |  | x |  |  | 0.545 (0.457, 0.632) |
| Medial JWSL ≥ 0.70mm | x |  | x |  |  | 0.559 (0.496, 0.621) |
| Lateral JWSL ≥ 0.70mm | x |  | x | x |  | 0.532 (0.447, 0.617) |

*no biomarkers selected

# Table S8. Results of penalized logistic regression to predict outcomes from serum and urine biomarkers (covariates not included) (n=681).

| **Outcome** |  |  |  |  | **Biomarkers** | | | | | **AUC** |
| --- | --- | --- | --- | --- | --- | --- | --- | --- | --- | --- |
|  | **sCTX-I** | **sHA** | **sNTX-I** | **sPIIBNP (pro-C2)** | **uCTXIα** | **uCTXIβ** | **uC2C-HUSA** | **uCTXII** | **uNTXI** |  |
| *Non-creatinine normalized* | | | | | | | | | | |
| JWSL ≥ 0.70mm |  | x |  |  |  |  | x |  |  | 0.581 (0.526, 0.636) |
| JWSL ≥ 0.50mm |  |  | x |  |  |  | x |  |  | 0.593 (0.547, 0.639) |
| Pain progression |  |  | x |  |  |  |  |  |  | 0.558 (0.509, 0.608) |
| JWSL ≥ 0.70mm + pain progression |  | x |  | x |  |  |  |  |  | 0.585 (0.495, 0.675) |
| Medial JWSL ≥ 0.70mm |  | x |  |  |  |  | x |  |  | 0.576 (0.513, 0.640) |
| Lateral JWSL ≥ 0.70mm |  | x |  |  |  |  | x |  |  | 0.567 (0.475, 0.659) |
| *Creatinine normalized* | | | | | | | | | | |
| JWSL ≥ 0.70mm |  | x |  |  | x |  | x |  |  | 0.581 (0.525, 0.638) |
| JWSL ≥ 0.50mm |  | x | x |  | x |  | x |  |  | 0.568 (0.522, 0.615) |
| Pain progression |  |  | x |  |  |  |  |  |  | 0.558 (0.509, 0.608) |
| JWSL ≥ 0.70mm + pain progression |  | x |  | x |  |  | x |  |  | 0.613 (0.528, 0.699) |
| Medial JWSL ≥ 0.70mm |  | x |  |  | x |  |  |  |  | 0.566 (0.498, 0.633) |
| Lateral JWSL ≥ 0.70mm^2^ |  | x |  | x |  |  | x |  |  | 0.531 (0.439, 0.623) |

# Table S9. Cross-Validated AUCs for models with covariates only.

|  | **Cohort** | | |
| --- | --- | --- | --- |
| **Outcome** | **Serum**  **(n=722)** | **Urine**  **(n=786)** | **Combined**  **(n=681)** |
| JWSL ≥ 0.70mm | 0.605 (0.552, 0.658)  BMI, KLG | 0.603 (0.554, 0.652)  BMI | 0.612 (0.557, 0.667)  BMI, KLG |
| JWSL ≥ 0.50mm | 0.598 (0.552, 0.643)  BMI | 0.608 (0.565, 0.651)  BMI | 0.607 (0.560, 0.654)  BMI |
| Pain progression | 0.494 (0.447, 0.541)  Age, BMI, KLG | 0.487 (0.441, 0.533)  Sex | 0.492 (0.442, 0.543)  Age |
| JWSL ≥ 0.70mm + pain progression | 0.564 (0.483, 0.645)  BMI | 0.550 (0.469, 0.630)  BMI | 0.568 (0.481, 0.656)  BMI, age |
| Medial JWSL ≥ 0.70mm | 0.604 (0.544, 0.663)  BMI, KLG, sex | 0.590 (0.534, 0.646)  BMI, Sex | 0.623 (0.560, 0.685)  BMI, KLG, Sex |
| Lateral JWSL ≥ 0.70mm | 0.564 (0.473, 0.655)  BMI | 0.585 (0.497, 0.674)  BMI | 0.580 (0.485, 0.675)  Age, BMI |
